# Path Optimization along Buoys Based on the Shortest Path Tree with Uncertain Atmospheric and Oceanographic Data

**DOI:** 10.1155/2021/6663446

**Published:** 2021-02-27

**Authors:** Han Xue, Tian Chai

**Affiliations:** School of Navigation, Jimei University, Xiamen 361021, Fujian, China

## Abstract

In order to design an optimized route for ships in line with economic benefits; avoid bad weather; reduce unnecessary detours; shorten the navigation time; and achieve the purpose of safety, fuel saving, and punctual arrival, this paper takes the navigation mark as the node of the tree, takes the connection of the adjacent navigation marks as the tree path, and divides the distance of the adjacent buoys by the ship's speed as the path cost. The speed calculation collects the current hydrometeorological data such as wind and wave data, uses Aertssen's deceleration formula to adjust the speed, and improves Dijkstra to find the shortest path. In the experiment, two routes from Dongdu to Xiamen Gang Kou are compared under bad weather conditions. Route 1 is with 5.877 m/s average wind speed, 0.860 m/s wave speed, and total distance 34717 m. Route 2 is with 8.503 m/s average wind speed, 1.429 m/s wave speed, and total distance 30223 m. The calculated ship speed travelling in route 1 is 12.243 km, and its travelling time is 1.53 h. The calculated ship speed travelling in route 2 is 10.523 km, and its travelling time is 1.55 h. Although the total distance of route 1 is longer, it takes less time for ships to travel in route 1. The experimental results verify the effectiveness of the navigation algorithm based on the shortest path tree of uncertain weather maps.

## 1. Introduction

Ships are often affected by a variety of different marine meteorological factors. The ship route selection is often not satisfactory. Therefore, it is of great significance and economic effect to design an optimized route for ships and avoid bad weather, so as to shorten the voyage time and achieve the purpose of safety, fuel saving, and punctual arrival at the port.

In the research of the shortest path tree, Li proposed a graph-based decomposition dynamic shortest path tree algorithm [1]. An actual network topology was used to calculate the convergence time of static algorithm and various dynamic algorithms when the weight of a single edge changed. Based on the dynamic shortest path tree algorithm, Wang proposed an efficient shortest path tree maintenance algorithm. Through statistical analysis of the network operation in a certain period of time, the algorithm obtained the edges with frequent changes in weights and dealt with the edges with frequent changes in weights [2]. Through the statistical analysis of the operation of the network in a certain period of time, the edge with frequent weight changed, so as to avoid adding it to the shortest path tree and reducing the update times of the shortest path tree. Dai improved the reliability of the shortest path tree by constantly replacing the edges with high probability and then obtained the most reliable shortest path tree [3]. A new calculation method was proposed to calculate the reliability of the shortest path tree. Yang proposed unstable edge and statistical unstable edge [4]. It could make the constructed path tree more stable on the dynamic network and spend less operation to update the shortest path tree.

In the study of meteorological routes, Wen proposed an intelligent hybrid algorithm to design the objective function according to the navigation requirements [5]. The objective function and fitness function were designed according to the navigation requirements and multiple constraints. Li proposed the optimization of ship meteorological route and built a weighted punishment fitness evaluation model based on the influence factors of weather and sea state [6]. By optimizing the route and speed, the weather routes under various user preferences could be obtained. Zhang et al. optimized the dynamic optimal meteorological route according to the medium and short-term forecast [7]. The route according to the medium and short-term forecast data was dynamically selected. Using risk analysis theory and technology, Sun can objectively and quantitatively simulate and evaluate the safety of the routes that may reach the target sea area within two days [8]. The quantitative evaluation of the route where ships will arrive in two days was carried out.

In the navigation research of navigation marks to navigation, Sang obtained the navigation mark in the route obtained from the database [9]. The third-party radar chart data and the route radar map and the third-party radar map were superimposed to form the route radar map. The meteorological data on the route weather radar map is updated in real time [10]. The proposed route weather radar chart showed a visual platform for passengers to query the route weather. Lin and Zhong proposed an intelligent optimization design of ship routes based on the information data of ports, channels, meteorology, and hydrology [11]. The database of intelligent routes was developed to provide ships with instant information of marine environment.

Due to the uncertainty and inaccuracy of environmental data, fuzzy logic is a good tool to deal with environmental data. Cacciola et al. derived a representative model of the multivariate relationships and predicted in order to organize traffic in advance from the estimated parameters [12]. Cacciola et al. provided exhaustive details on how to build a fuzzy system [13]. Postorino and Versaci explained in detail how to easily structure a bank of fuzzy rules [14]. Structuring fuzzy or neuro-fuzzy rule banks was capable of modeling the problem of uncertainty and imprecision present in the data.

In this paper, the navigation mark is taken as the node of the tree, the connecting line of the navigation mark in the channel is taken as the path of the tree, and the distance of the adjacent beacon divided by the speed of the ship is taken as the path cost. The speed calculation collects the current hydrometeorological data such as wind and wave data, uses Aertssen's deceleration formula to adjust the speed, and improves Dijkstra to find the shortest path.

## 2. Shortest Path Tree of Uncertain Weather Map

### 2.1. Aertssen's Deceleration Method

Aertssen proposed the allowable speed of cargo ship based on propeller idling, deck wave, bottom beating, and cargo moving [15]:

(1) $$\begin{array}{c} \frac{U_{ws}}{U_{s}}=1-\frac{m}{L_{pp}}-n, \end{array}$$

where *U*_*ws*_ is the allowable speed, *U*_*s*_ is the still water velocity, *L*_*pp*_ is the length between the two columns, and *m* and *n* are the stall estimation formula coefficients.  Table 1 shows the coefficient of ship stall estimation formula.

### 2.2. Dijkstra Shortest Path

Dijkstra algorithm is a typical single source shortest path algorithm, which is used to calculate the shortest path from one node to all other nodes [16]. It extends from the starting point to the outer layer until it reaches the end point. In the adjacency matrix of a given graph, the shortest path is recorded by traversing all the paths of the known graph, and then the replacement is continuously judged in the loop. Firstly, the set of all points is divided into two parts: one side has been traversed, and the other side has not been traversed. Firstly, the initial value is given to infinity, and then all nodes are traversed to find the shortest path with the smallest weight in the traversal path. The shortest path can be obtained through constant modification. (Algorithm 1)

The computation cost of the algorithm is O(*V*log*V*).

### 2.3. Proposed Algorithm

In this algorithm, the navigation mark is used as the node of the tree, the connecting line of the navigation mark in the channel is taken as the path of the tree, and the distance of the adjacent navigation mark divided by the ship speed is the path cost. Speed calculation collects current hydrometeorological data such as wind and wave data and adjusts the speed by Aertssen's deceleration formula.

The specific algorithm is described as follows:

  Step 1: take the navigation mark as the vertex set *V* of the tree, and take the connecting line of the adjacent navigation marks of the channel as the path edge set *E* of the tree. A nonnegative weight simple connected undirected graph *G* = <*V*, *E*> is constructed. *D* is the adjacency matrix of graph *G*. The starting point is P0.
  Step 2: assign initial values *D* [*i*] = inf (infinity) and mark [*i*] = 0.
  Step 3: read the hydrometeorological data such as wind and wave of the current channel.
  Step 4: according to formula (1), calculate the speed and time cost of ships passing through each path, where *D* is the adjacency matrix of graph *G*.
  Step 5: if *v* makes *d* [*J*] smaller, that is, *d* [*v*] + *L* [*v*] [*J*] < *d* [*J*], update *d* [*J*], that is, *d* [*J*] = *D* [*v*] + *L* [*v*] [*J*] and update path, that is, path [*J*] = *v*.
  Step 6: select the nodes with the minimum path value and put them into the set.
  Step 7: if the remaining node set is empty, end the output structure; otherwise, go to step 3.

## 3. Experiments

### 3.1. Instance Introduction

There are two routes from Dongdu to Xiamen Gang Kou. The first route is listed in Table 2 with the navigation marks passed by.

The second route is listed in Table 3 with the navigation marks passed by.

This algorithm is implemented on the cruise ship 16301 as an example.  Table 4 shows the basic information of the ship.

### 3.2. Path Optimization under Good Weather Conditions

**Figure 1:**
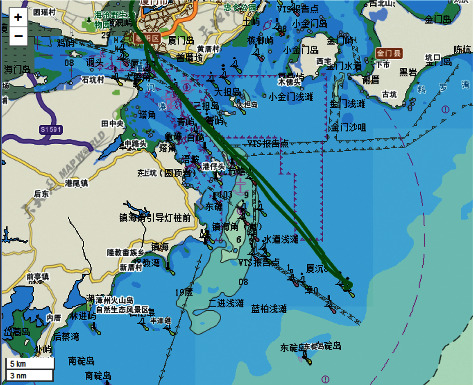
Recommended route from Dongdu to Koumen.

Under good weather conditions, the recommended route from Dongdu to Koumen is the second route as shown in Figure 1.

**Figure 2:**
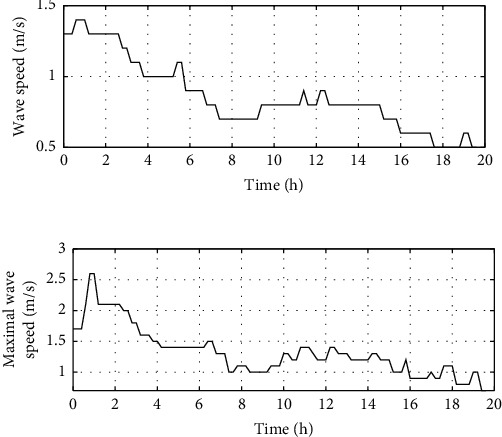
Wind speed in good weather.

**Figure 3:**
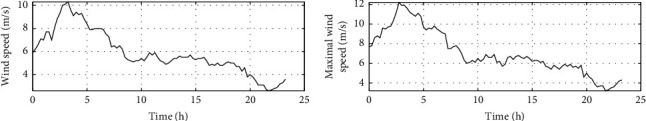
Wave speed in good weather.

Under good weather conditions, the wind speed curve is shown in Figure 2 and the wave speed curve is shown in Figure 3 [17].

**Figure 4:**
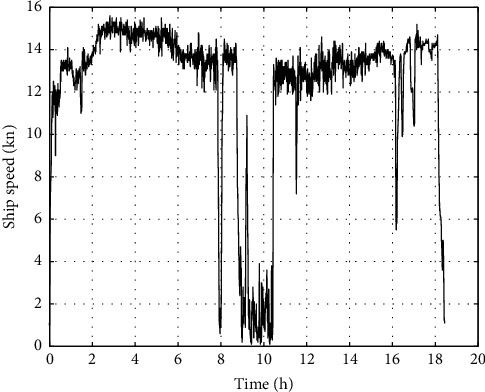
Ship speed in good weather.

Under good weather conditions, the calculated ship speed is shown in Figure 4.

### 3.3. Path Optimization under Bad Weather Conditions

**Figure 5:**
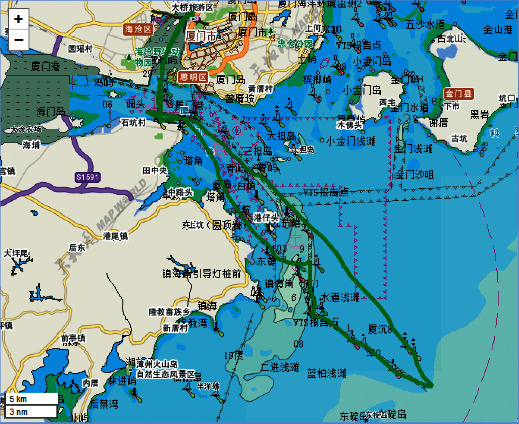
Recommended route from Dongdu to Koumen under bad weather.

Under bad weather conditions, the recommended route from Dongdu to Koumen is the second route as shown in Figure 5.

**Figure 6:**
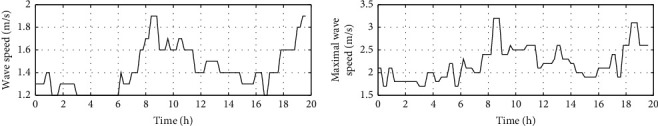
Wind speed in good weather.

**Figure 7:**
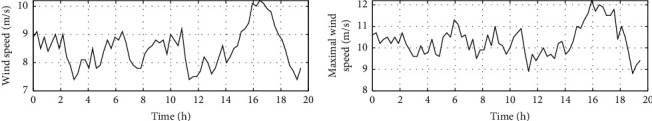
Wave speed in good weather.

Under bad weather conditions, the wind speed curve is shown in Figure 6 and the wave speed curve is shown in Figure 7.

**Figure 8:**
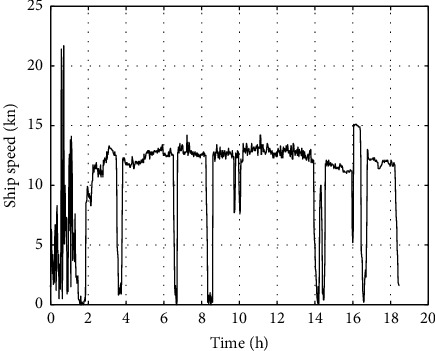
Ship speed in good weather.

Under good weather conditions, the calculated ship speed is shown in Figure 8.

### 3.4. Comparison of Meteorological Routes

The calculation results under bad weather condition are compared in Table 5.

From Table 5, we can see that, under bad weather condition, the wind and wave speeds in route 1 are slow. The ship navigation in route 1 is fast. The wind and wave speed in route 2 are fast. The ship navigation in route 1 is slow. Although the total distance of route 1 is longer, it takes less time for ships to travel in route 1.

## 4. Conclusion

In this paper, the navigation mark is taken as the node of the tree, the connecting line of the navigation mark in the channel is taken as the path of the tree, and the distance of the adjacent beacon divided by the speed of the ship is taken as the path cost. The speed calculation collects the current hydrometeorological data such as wind and wave data, uses Aertssen's deceleration formula to adjust the speed, and improves Dijkstra to find the shortest path. In the experiment, two routes from Dongdu to Xiamen Gang Kou are compared under bad weather conditions. Route 1 is with 5.877 m/s average wind speed, 0.860 m/s wave speed, and total distance 34717 m. Route 2 is with 8.503 m/s average wind speed, 1.429 m/s wave speed, and total distance 30223 m. The calculated ship speed travelling in route 1 is 12.243 km and its travelling time is 1.53 h. The calculated ship speed travelling in route 2 is 10.523 km and its travelling time is 1.55 h. Although the total distance of route 1 is longer, it takes less time for ships to travel in route 1. The experimental results verify the effectiveness of the navigation algorithm based on the shortest path tree of uncertain weather maps.

This paper will continue to optimize the algorithm in the future. Fuzzy logic will be used to model the uncertainty of data. Tide height and foggy weather conditions will be considered. Dijkstra algorithm will be improved.

## Figures and Tables

**Algorithm 1:**
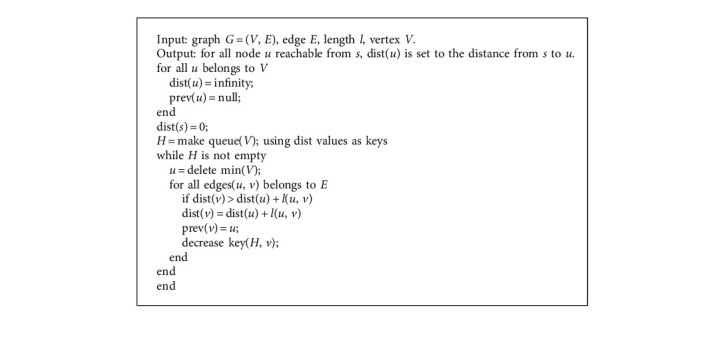
Dijkstra shortest path algorithm.

**Table 1:** Coefficient of ship stall estimation formula.

	Beaufort scale	5	6	7	8
Head sea	*m*	9	13	21	36
	*n*	0.02	0.06	0.11	0.18

Bow sea	*m*	7	10	14	23
	*n*	0.02	0.05	0.08	0.12

Beam sea	*m*	3.5	5.0	7.0	10.0
	*n*	0.01	0.03	0.05	0.07

Following sea	*m*	1	2	4	7
	*n*	0	0.01	0.02	0.03

**Table 2:** The first route.

No.	Longitude	Latitude	Distance between adjacent buoys (m)
404	118°04.0′E	24°24.8′N	1864
Q2-1	118°04.5′E	24°23.9′N	4364
Q1-1	118°06.4′E	24°22.3′N	8265
106	118°08.8′E	24°18.4′N	1781
104	118°09.7′E	24°17.9′N	1643
102	118°10.3′E	24°17.2′N	5897
8	118°13.7′E	24°16.5′N	3287
6	118°14.9′E	24°15.1′N	7616
Xiamen Gang Kou	118°17.6′E	24°11.8′N	—

**Table 3:** The second route.

No.	Longitude	Latitude	Distance (m)
404	118°04.0′E	24°24.8′N	1196
402	118°04.7′E	24°24.7′N	1461
22	118°05.5′E	24°24.4′N	1541
20	118°06.3′E	24°24.8′N	1854
Q2	118°06.4′E	24°23.8′N	1792
18	118°07.0′E	24°23.0′N	2503
16	118°08.0′E	24°22.0′N	1477
14-1	118°08.0′E	24°21.2′N	1620
14	118°08.9′E	24°20.9′N	4242
12	118°10.7′E	24°19.3′N	1480
Xiachen No. 7	118°16.5′E	24°13.3′N	875
Xiachen No. 8	118°16.1′E	24°13.6′N	4182
Xiamen Gang Kou	118°17.6′E	24°11.8′N	—

**Table 4:** Ship information.

Parameter	Value
Call sign	BTTD
IMO	402657927
Ship length	32 m
Breadth	6 m
Ship name	Cruise ship 16301
MMSI	413045130

**Table 5:** Information of different routes under bad weather condition.

	Avg. wind speed (m/s)	Avg. wave speed (m/s)	Avg. ship speed (km)	Total distance (m)	Travelling time (h)
Route 1	5.877	0.860	12.243	34717	1.53
Route 2	8.503	1.429	10.523	30223	1.55

## Data Availability

The data used to support the findings of this study are included within the article.

## References

[1] Li X. (2011). *Research on Dynamic Algorithm of Shortest Path Tree*.

[2] Wang S. X. (2017). *Research on Shortest Path Tree Algorithm in Dynamic Network*.

[3] Dai L. W. (2017). *Research of the Shortest Path Tree on Uncertain Graph*.

[4] Yang X. H. (2016). *Analysis and Research of Shortest Path Tree Algorithm in Dynamic Environment*.

[5] Wen T. (2019). Intelligent hybrid algorithms for meteorological route planning under multiple constraints. *Journal of Ship Design*.

[6] Li M. F., Wang S. Z., Xie Z. X. (2020). Multi-variable-multi-objective optimization of ship routes under rough weather condition. *Navigation of China*.

[7] Zhang X. C., Cui J. H., Rong L. (2014). Design and selection of optimal weather route. *Tianjin Navigation*.

[8] Sun C. Z., Ding D. W., Liu D. G. (2014). Auxiliary decision-making simulation based on meteorological and hydrologic information for naval navigation security. *Journal of Dalian Maritime University*.

[9] Li H. (2017). Application of meteorological navigation in ship route design. *Navigation Technology*.

[10] Sang L., Xuan T., Zheng H. F. (2015). A method and system for obtaining route weather radar chart.

[11] Lin S. Y., Zhong T. (2018). Discussion on the relationship between intelligent ship and intelligent navigation insurance. *Science and Technology Innovation Herald*.

[12] Cacciola M., Pellicanò D., Megali G., Lay-Ekuakille A., Versaci M., Morabito F. C. Aspect about air pollution prediction on urban environment.

[13] Cacciola M., Calcagno S., Morabito F. C., Versaci M. (2007). Swarm optimization for imaging of corrosion by impedance measurements in eddy current test. *IEEE Transactions on Magnetics*.

[14] Postorino M. N., Versaci M. (2014). A geometric fuzzy-based approach for airport clustering. *Advances in Fuzzy Systems*.

[15] Aertssen G. Speed loss due to weather on one class of container ship in the north Atlantic with reference to relationship between wind and waves.

[16] Dijkstra E. W. (1959). A note on two problems in connexion with graphs. *Numerische Mathematik*.

[17] China Ocean Forecast Network [J/OL], http://www.oceanguide.org.cn

